# Estimating the Size of the Female Sex Worker Population in Kenya to Inform HIV Prevention Programming

**DOI:** 10.1371/journal.pone.0089180

**Published:** 2014-03-03

**Authors:** Willis Omondi Odek, George N. Githuka, Lisa Avery, Peter K. Njoroge, Lombe Kasonde, Marelize Gorgens, Joshua Kimani, Lawrence Gelmon, Gloria Gakii, Shajy Isac, Emmanuel Faran, Helgar Musyoki, William Maina, James F. Blanchard, Stephen Moses

**Affiliations:** 1 Centre for Global Public Health, Department of Community Health Sciences, University of Manitoba, Winnipeg, Canada; 2 National AIDS/STI Control Programme (NASCOP), Ministry of Health, Nairobi, Kenya; 3 School of Public Health, University of Nairobi, Nairobi, Kenya; 4 International Bank for Reconstruction and Development, Washington, District of Columbia, United States of America; 5 Department of Medical Microbiology, University of Manitoba, Winnipeg, Canada; International AIDS Vaccine Initiative, United States of America

## Abstract

**Background:**

The high burden of HIV infections among female sex workers (FSW) in sub-Saharan Africa has been long recognised, but effective preventive interventions have largely not been taken to scale. We undertook a national geographical mapping exercise in 2011/2012 to assess the locations and population size of FSW in Kenya, to facilitate targeted HIV prevention services for this population.

**Methods and Findings:**

We used a geographical mapping approach, consisting of interviews with secondary key informants to identify “hot” spots frequented by FSW, their operational dynamics and the estimated numbers of FSW in those spots. This was followed by validation of the estimates through interviews with FSW at each spot identified. The mapping covered Nairobi, the capital city of Kenya, and 50 other major urban centres. In total, 11,609 secondary key informant interviews were conducted to identify FSW spots. Further, a total of 6,360 FSW were interviewed for spot validation purposes. A total of 10,670 spots where FSW congregate were identified. The estimated FSW population in all the towns mapped was 103,298 (range 77,878 to 128, 717). Size estimates in the towns mapped were extended to smaller towns that were not mapped, using a statistical model. The national urban FSW population estimate was 138,420 (range 107, 552 to 169, 288), covering all towns of over 5,000 population. We estimated that approximately 5% of the urban female population of reproductive age in Kenya could be sex workers, which is consistent with previous estimates from other sub-Saharan African countries.

**Conclusions:**

This study provides the first national level data on the size of the FSW population in Kenya. These data can be used to enhance HIV prevention programme planning and implementation for FSW, to form the basis for impact evaluations, and to improve programme coverage by directing efforts to locations with the greatest need.

## Introduction

In Kenya, as in many parts of sub-Saharan Africa, female sex workers (FSW) bear the greatest burden of HIV infection. As early as 1985, a study reported that HIV prevalence was as high as 61 per cent among a group of FSW in Nairobi [1]. Pooled HIV prevalence data available shows that three decades into the HIV response, nearly half (45.1% - range 44.0 to 46.2) of FSW in Kenya are infected with HIV, compared to 7.7 per cent of the general female population [2]. The development of effective interventions among FSW requires accurate information on where they are based and their population size. The common approaches to FSW population size estimation are census and enumeration, capture-recapture and multiplier methods [3]. Low coverage of targeted interventions for FSW and the quality of programme data are common challenges to the use of the service multiplier method for population size estimation. The application of both the census and enumeration methods can be hampered by the hidden nature of FSW. The capture-recapture method is widely used, but this method is based on complex assumptions that may not always be applicable in FSW populations [4]–[6]. Perhaps most importantly, methods other than census and enumeration provide population size estimates with limited practical information to guide HIV prevention programming.

FSW population size estimation exercises have been undertaken over the past few years in some towns in Kenya, including Nairobi, Mombasa and Kisumu cities [7]–[9], using the methods indicated above, but there have been large gaps in their coverage. Both the Government of Kenya and prevention programme implementers agreed that there was a critical need to develop a better understanding of locations and size estimates of FSW and other high-risk populations throughout the country to inform HIV prevention programming. A geographical mapping approach was deemed the most appropriate one to provide data that could be used for coordinated and integrated programme implementation and roll-out. The choice of the geographical mapping and size estimation approach was based on experiences in other countries in Asia where this method has been used, and results applied to develop and improve targeted HIV prevention programmes for high-risk populations [10]–[12]. The overall goal of the mapping exercise was to provide accurate information on the locations and size of FSW and other high-risk populations for HIV in key urban and semi-urban areas of Kenya, with a view to improving the scale, coverage, and aligned roll-out of HIV prevention programmes among these populations. We report here on the results of this exercise for female sex workers.

## Methods

### Ethics statement

This study involved interviews with secondary key informants (KIs) found at a variety of public places, to identify spots or places frequented by FSW in their locality and estimated numbers of FSW in those spots, followed by validation of the estimates through interviews with FSW at each of the identified spots. The data collection teams were accompanied by FSW peer educators who provided background information on the study and helped with the identification of FSW respondents. The interviewers explained to both secondary KIs and FSW that their participation in the study was fully voluntary, the study did not pose any risks to them and the data collected would be kept strictly confidential and used for the research purposes only. No personal identifying information was collected from both the secondary KIs and FSW as part of the study. Both the secondary KIs and FSW interviewed provided verbal consent because sex work is illegal in Kenya, posing a challenge to written consent. Moreover, FSW in the study were identified from diverse hotspots, including noisy bars and poorly-lit streets, which were not amenable to a written consenting process. The interviewers signed on the data collection forms to confirm that they had obtained verbal consent for both secondary key informants and FSW interviews. These study procedures were reviewed and approved by the Kenyatta National Hospital Ethical Review Committee, Nairobi, Kenya.

### Methodological approach

Female sex workers were defined as ‘women who exchange anal, vaginal and/or oral sex for money or other items of value, primarily with men’ [13]. Sex work spot typologies that are defined in the *Kenya National Guidelines for HIV/STI Programmes for Sex Workers*, namely, street-based, home-based, venue-based, road (truck stop)-based, sex den-based, massage parlour-based and escort services-based sex work were examined in the study [13].

A geographical mapping approach, which identifies the key locations where FSW can be found and enumerated was used for the study. The approach was based on programmatic experience in Asia, which has shown that populations at an increased risk for HIV acquisition and transmission generally congregate and/or meet clients (or casual partners) in specific geographic settings. Studies in sub-Saharan Africa have also pointed to geographical clustering of HIV infections and risk behaviours, underlying the importance of identifying such locations and the number of FSW linked to the locations for targeted HIV preventive interventions [14], [15]. The geographical mapping approach focuses on identifying the locations frequented by high-risk populations, and characterises specific spots in terms of operational typologies and the sexual networks present [10]–[12]. Preliminary steps of the geographical mapping approach involve developing or acquiring maps of the targeted area, segmentation of the target area into smaller geographic zones to facilitate field work planning and data collection, and identifying and enlisting the support of key stakeholders and gatekeepers linked to the spots and the target areas. Data on the locations where populations most-at-risk for HIV congregate were collected in two sequential steps, described as Level 1 and Level 2 activities.

#### Level 1

In the first level activity, information was gathered systematically from carefully selected secondary key informants (KIs) regarding locations or spots (“hot spots”) where FSW congregate or meet casual or paying sexual partners. The secondary KIs were people considered to be knowledgeable about their local area, and were identified from a variety of public places such as at taxi ranks, bus stops, fuel stations, shopping malls, streets, bars, and other workplaces. To facilitate Level 1 data collection, each targeted area was divided into smaller geographic zones based on population estimates, administrative boundaries, knowledge of local programme implementers on areas where FSW may be found and/or other physical features and landmarks. The zoning ensured complete coverage of targeted urban centres. The division of the targeted urban centres into smaller geographic zones was done jointly with the Ministry of Health's Provincial and District AIDS Coordinators. Interviews were conducted with about 60 secondary key informants from across the smaller geographic zones to identify spots frequented by FSW within each of the zones. This sample size per zone was considered sufficient to achieve information saturation on spots frequented by FSW within each smaller geographic zone. At this stage, a spot was considered active even if only one or a small number of FSW frequented it. The secondary key informants provided the physical addresses of such spots, along with their estimated minimum and maximum number of FSW that frequented the spots. The output of Level 1 activity was a comprehensive list of unique spots where FSW might be found, the typology of the spot (for example, bar, street, massage parlour, among others), operational dynamics of each spot (for example, peak and non-peak times), and estimated minimum and maximum number of FSW at each spot.

#### Level 2

The second stage involved visits to the spots identified at Level 1 for interviews with FSW themselves, to validate the existence of the spots, to ascertain whether or not spots were in fact frequented by FSW (that is, if spots were active or inactive), to ascertain the operational characteristics of the spots, and to obtain estimates of the minimum and maximum number of FSW who frequented the active spots. In this validation stage, spots that were mentioned by the least number of secondary key informants in Level 1 were given priority, because these were the most likely to have been incorrectly identified. FSW peer educators were identified through existing FSW programmes in each selected urban centre, where they were available, to accompany the study team to the identified spots and mobilise individual FSW for interviews on estimated FSW population at the spots. Each provincial administration provided a letter of authorisation for the study, and in some cases, police security to the study team, as FSW interviews were usually conducted at night. Data generated through these primary key informant interviews were used in generating population size estimates, while for those spots not re-visited, an average of the estimates from secondary key informants in Level 1 were used.

### Study sites

**Figure 1:**
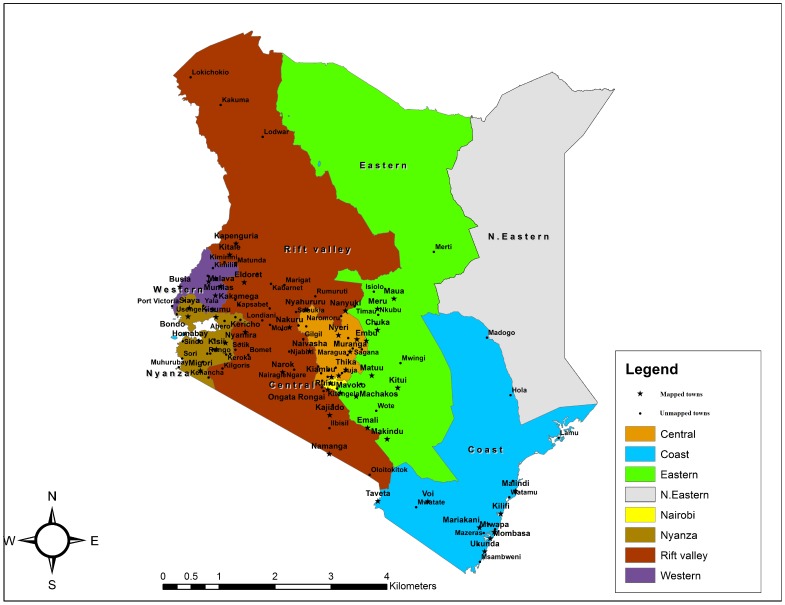
The spatial distribution of the towns covered for the geographical mapping study. This figure shows the spatial distribution of the towns covered for the geographic mapping study vis-à-vis those not covered within each administrative province in Kenya.

The geographic mapping study covered seven out of the eight former administrative provinces in Kenya, namely: Nairobi, Central, Eastern, Nyanza, Western, Coast and Rift Valley Province. North Eastern Province, situated near the border of Kenya and Somalia, was excluded because of security concerns. The whole of Nairobi City was mapped, and within each of the other provinces, at least seven major towns and municipalities were selected in consultation with the Ministry of Health's National AIDS/STI Control Programme (NASCOP), the National AIDS Control Council (NACC), FSW programme implementers, the United Nations family, development partners, and other stakeholders. The towns selected were usually headquarters of the former districts, most of which are now County headquarters in Kenya's new administrative structure. The population of the urban centres selected for mapping outside of Nairobi City represented 72 per cent of the total population of towns with over 5,000 population in each province (Table 1). Because of time and resource constraints, it was not possible to include the remaining towns with over 5,000 population. FSW enumeration was not undertaken in towns with less than 5,000 population or in rural areas. Our assumption for this mapping study was that most commercial sex occurs in larger centres (i.e. in towns with over 5,000 population). Together with Nairobi City, 51 urban centres were included in this study. Figure 1 shows the spatial distribution of the towns that were mapped and those not mapped within each province.

**Table 1:** Population of towns with over 5,000 population and mapping coverage, by province.

Province	Population of towns with 5,000+ population^a^			% population of towns mapped
	Mapped	Not mapped	Total	
Central	585,526 (7)	409,355 (5)	994,881 (12)	59%
Coast	1,213,334 (8)	60,191 (5)	1,273,525 (13)	95%
Eastern	273,641 (10)	116,330 (2)	389,971 (12)	70%
North Eastern	0	268,804 (7)	268,804 (7)	0%
Nyanza	450,998 (7)	98,715 (6)	549,713 (13)	82%
Rift Valley	894,444 (11)	467,867 (15)	1,362,311 (26)	66%
Western	281,265 (7)	41,115 (1)	322,380 (8)	87%
**Total**	**3,699,208 (50)**	**1,462,377 (41)**	**5,161,585 (91)**	**72%**

a Number of towns shown in parentheses.

### Data collection and quality assurance

This study was implemented in collaboration with five organisations in Kenya that were implementing HIV prevention programmes among FSW in different regions of the country at the time that the study was planned, namely, the Kenya AIDS Control Project, Universities of Manitoba and Nairobi (KACP); the Centre for HIV Prevention and Research, University of Nairobi (CHIVPR); Impact Research and Development Organisation (IRDO); Hope Worldwide Kenya (HWWK); and the International Centre for Reproductive Health, Kenya (ICRH-K). Key selection criteria for these organisations included long experience implementing programmes with FSW and other high-risk populations within the region and research experience. These organisations were also included to build their capacity in the geographical mapping methodology and to help facilitate the use of the enumeration data for HIV prevention programming in the country. The mapping study was not restricted to the sites served by the implementing partners, but covered all areas within the urban centres that were selected. Training on the geographic mapping methodology was conducted for field team supervisors, data managers and study site coordinators from each implementing partner; they in turn trained their field teams. The study technical team, comprising local University of Manitoba, University of Nairobi and NASCOP staff also supported the collaborating organisations during training of their data collection teams. Field data collection lasted from November 2011 to February 2012.

The data collected at Level 1 were edited by the fieldwork team to standardise names of spots, and hence reduce duplication. Throughout the data collection process, local University of Manitoba, University of Nairobi and NASCOP staff undertook field monitoring and quality assurance visits to ensure that the minimum number of secondary key informant interviews within each of the smaller geographic zones were met and that all spots identified for validation were visited and FSW interviewed at those spots. The supervisory visits also identified any security and field-access related challenges faced by the study team and addressed these with the local administration, NASCOP's District AIDS Coordinators and local HIV prevention programme managers. Field monitoring visits were conducted with each field team at least once during Level 1 data collection and at least twice during Level 2 data collection.

### Data analysis

Data were processed using a Microsoft Access database with in-built quality checks. The same software was used to generate a list of unique spots and calculate FSW population size estimates by spot typology, town and province. The FSW interviewed at the identified spots for validation purposes were also asked about their mobility across spots. This information on mobility was used to adjust the population size of FSW, thereby reducing double-counting of FSW frequenting multiple spots. The adjustment for mobility was done using a mathematical model, expressed in Equation 1.
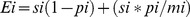
(1)Where E*i* is the estimated number of FSW in a town, *si* is the estimated number of FSW at a spot level, *pi* is the proportion of FSW soliciting clients in more than one spot and *mi* is the mean number of spots from which a FSW solicits clients. The analysis provided minimum and maximum estimates for each spot, urban centre and province. To arrive at a point estimate, averages of the minimum and maximum estimates were calculated.

Province-wide FSW population size estimates were made through regression modelling from the towns mapped, taking into consideration the population coverage of the towns of over 5,000 population mapped vis-à-vis those towns of over 5,000 population not mapped, within each province. The assumption for the regression model was that there exists a relationship between the adult population and estimated FSW population, and that the regression model can be used to examine the strength of this relationship. Therefore, if the adult population of a mapped town is regressed on a standardized variable, the regression coefficient can be used to estimate the expected number of FSW for the adult population of a given town. We used FSW per 1000 adults as the standardized dependent variable and the adult population size as the independent variable (Equation 2).
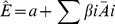
(2)Where Ê is the estimated FSW population per 1000 adult population in a given town, *a* is the constant of the regression model, *βi* is the coefficient for the variable (adult population), and *Ai* is the adult population. Since this relationship need not always be linear, we also included adult population square as an independent variable along with adult population (Equation 3).
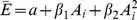
(3)Where 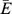 is the estimated FSW population per 1000 adult population in a given town, *a* is the constant of the regression model, *β_1_* is the coefficient for the variable (adult population), *β_2_ is* the coefficient for the variable adult population square and *Ai* and A_i_^2^ are the adult population and its square, respectively. The model coefficient and the mean of the coefficient were used in a multiple classification analysis [16] to arrive at the estimated number of FSW for a given adult population. The standard error (SE) of the estimate was used to estimate lower and upper population estimate bounds. Thus, A(−2 SE) and A(+2 SE) were considered as the lower and upper limits of the estimates, respectively.

The estimates derived from the regression model for the towns mapped were similar to those obtained from the mapping itself, hence we used the mapping estimates as the final estimates for these towns. The regression model estimates were used as the final estimates for all the other towns that were not mapped. In this way, the FSW population of North Eastern Province, which was not mapped, was extrapolated from the average national estimates of FSW in towns of over 5,000 population, from the other seven provinces.

## Results

### Key informant interviews

**Figure 2:**
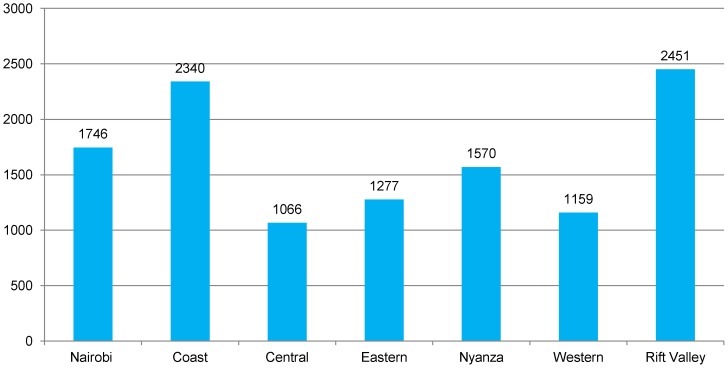
Total number of secondary key informant interviews, by province. The figure shows the total number of secondary key informants interviewed within each province to identify spots frequented by FSW.

A total of 11,609 secondary key informants were interviewed to identify spots frequented by FSW, with more than 1,000 key informants per province (Figure 2). The secondary key informants were predominantly male (75%), and 53 per cent of them had secondary or higher level of education. A total of 6,360 FSW were interviewed for spot validation purposes. The mean age of the FSW interviewed was 27.5 (SD 6.3) years. The majority (95.7%) of the FSW interviewed had at least primary level education, while 41 per cent had secondary or higher level of education. Over one-half (57.5%) of FSW interviewed for spot validation purposes reported that they frequented more than one spot.

### Female sex worker spots

A total of 10,670 active FSW spots were identified through the mapping exercise, with about a quarter (24%) of these being in Nairobi City alone. Of the 2,539 active FSW spots identified in Nairobi, 21 per cent were located within Starehe district, which encompasses the Central Business District, while 14 per cent each were located in Embakasi and Kasarani districts, both large and densely populated residential areas (Table 2 and Table 3).

**Table 2:** Estimated number of active FSW spots and population sizes in the towns mapped in Nairobi, Central, Eastern, Coast and Nyanza provinces, Kenya.

Province	Town (or administrative units)	Estimated number of active FSW spots	% of total	Point estimate for FSW population	Estimated FSW population range (minimum to maximum)		% of total^a^
Nairobi	Dagoretti	274	11%	2,433	1,878	2,987	9%
	Embakasi	367	14%	3,878	2,817	4,939	14%
	Kamukunji	256	10%	2,491	1,973	3,008	9%
	Kasarani	366	14%	3,791	2,834	4,748	14%
	Lang'ata	277	11%	2,855	2,195	3,514	10%
	Makadara	238	9%	2,482	1,922	3,042	9%
	Starehe	530	21%	6,763	5,230	8,296	24%
	Westlands	231	9%	2,929	2,231	3,627	11%
*Sub-total*		*2,539*	*100%*	*27,620*	*21,081*	*34,160*	*100%*
Central	Nyahururu	83	14%	801	609	993	11%
	Nyeri	111	18%	988	758	1,217	13%
	Kerugoya Kutus	107	18%	739	604	874	10%
	Murang'a	54	9%	442	346	538	6%
	Thika	85	14%	1,933	1,385	2,481	26%
	Kiambu	61	10%	862	661	1,062	11%
	Ruiru	106	17%	1,808	1,380	2,235	24%
*Sub-total*		*607*	*100%*	*7,572*	*5,743*	*9,400*	*100%*
Eastern	Kitui	69	6%	794	561	1,026	8%
	Machakos	150	13%	872	632	1,112	8%
	Emali	54	5%	1,180	848	1,512	11%
	Matuu	52	5%	540	407	673	5%
	Mavoko	140	12%	1,973	1,334	2,611	19%
	Makindu	74	6%	786	599	973	7%
	Embu	196	17%	1,032	827	1,237	10%
	Meru	211	18%	1,276	1,060	1,492	12%
	Maua	119	10%	1,555	890	2,219	15%
	Chuka	90	8%	560	458	662	5%
*Sub-total*		*1,155*	*100%*	*10,567*	*7,616*	*13,517*	*100%*
Coast	Mombasa	774	55%	9288	6917	11660	56%
	Kilifi	69	5%	624	478	769	4%
	Mariakani	77	5%	624	478	771	4%
	Mtwapa	70	5%	1118	917	1319	7%
	Taveta	55	4%	489	378	600	3%
	Voi	59	4%	900	656	1144	5%
	Malindi	232	16%	2310	1788	2831	14%
	Ukunda	82	6%	1112	811	1413	7%
*Sub-total*		*1,418*	*100%*	*16465*	*12422*	*20508*	*100%*
Nyanza	Kisumu	534	42%	4,041	3,228	4,854	28%
	Bondo	104	8%	1,676	1,310	2,041	12%
	Siaya	67	5%	473	377	568	3%
	Kisii	215	17%	4,063	2,990	5,136	28%
	Migori	169	13%	2,272	1,698	2,846	16%
	Nyamira	93	7%	856	665	1,047	6%
	Homa Bay	85	7%	995	774	1,216	7%
*Sub-total*		*1,267*	*100%*	*14,375*	*11,042*	*17,708*	*100%*

a Based on point estimate for FSW population.

**Table 3:** Estimated number of active FSW spots and population sizes in the towns mapped in Western and Rift Valley provinces, Kenya.

Province	Town (or administrative units)	Estimated number of active FSW spots	% of total	Point estimate for FSW population	Estimated FSW population range (minimum to maximum)		% of total^a^
Western	Kakamega	206	11%	1,238	905	1,570	9%
	Webuye	232	13%	1,990	1,549	2,431	15%
	Bungoma	294	16%	1,994	1,540	2,448	15%
	Busia	257	14%	2,474	1,854	3,094	19%
	Mumias	317	17%	2,167	1,617	2,716	16%
	Vihiga	427	23%	2,749	2,031	3,467	21%
	Malaba	98	5%	708	554	862	5%
*Sub-total*		*1,831*	*100%*	*13,319*	*10,050*	*16,588*	*100%*
Rift Valley	Nakuru	339	18%	4,384	3,220	5,549	33%
	Naivasha	152	8%	925	568	1,282	7%
	Narok	159	9%	576	455	698	4%
	Kajiado	39	2%	282	226	338	2%
	Namanga	46	2%	345	325	365	3%
	Kapenguria	145	8%	1,004	766	1,241	8%
	Kitale	178	10%	815	635	994	6%
	Eldoret	429	23%	2,442	1,803	3,080	18%
	Nanyuki	92	5%	554	407	701	4%
	Kericho	125	7%	1,116	856	1,376	8%
	Ongata Rongai	149	8%	937	662	1,213	7%
*Sub-total*		*1,853*	*100%*	*13,380*	*9,923*	*16,837*	*100%*
**Grand Total**		**10,670**		**103,298**	**77,878**	**128,717**	

a Based on point estimate for FSW population.

### Female sex worker population estimates

The estimated population of FSW in all the towns mapped was 103,298 (range 77,878 to 128,717). Out of the 27,620 FSW in Nairobi City, a quarter were found in Starehe district, which encompasses the Central Business District, followed by Embakasi (14.0%) and Kasarani (13.7%) districts. In Coast province, the majority of FSW were in Mombasa city (9,288, 56%), followed by Malindi (2,310, 14%), Mtwapa (1,118, 7%) and Ukunda (1,112, 7%). In Nyanza province in western Kenya, the towns with the highest number of FSW were Kisumu city (4041, 28%), Kisii (4,063, 28%) and Migori (2,272, 16%). Nakuru town with an estimated 4,384 FSW accounted for 33 per cent of the FSW in Rift Valley province, followed by Eldoret (2,442, 18%), Kericho (1,116, 8%) and Kapenguria (1,004, 8%). In Western province, the majority of FSW were in Vihiga (2,749, 21%), Busia (2,474, 19%) and Mumias (2,167, 16%). Mavoko town accounted for 19 per cent (n = 1,973), while Maua (n = 1,555) and Meru (n = 1,276) towns accounted for 15 and 12 per cent, respectively, of the FSW in Eastern province. In Central province, Thika town had the largest number of FSW (1,933, 26%) followed by Ruiru (1,808, 24%) and Nyeri (988, 13%) towns (Table 2 and Table 3).

### Estimates of female sex workers by spot typology

**Figure 3:**
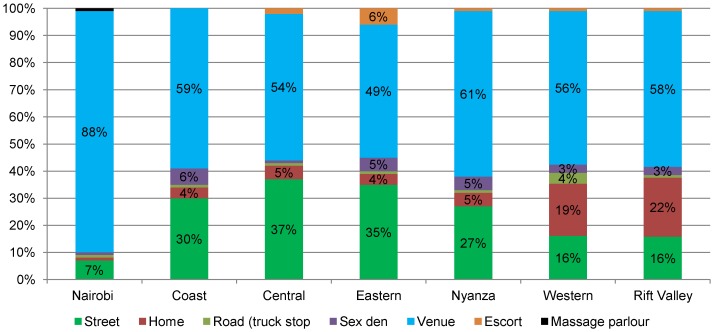
Proportional distribution of FSW population by typology of spot. The figure shows percentage distribution of estimated FSW population within each province by the types of spots from which they operate.

FSW were analysed further by the type of spot from which they operated. Street-based FSW as a proportion of all FSW was higher in Central (37%), Eastern (35%), Coast (30%) and Nyanza (27%) provinces than in Western (16%), Rift Valley (16%) and Nairobi (7%) provinces. Venue-based FSW were more common in Nairobi (88%) than in Nyanza (61%), Coast (59%), Rift Valley (58%), Western (56%), Central (54%) and Eastern provinces. Home-based FSW were more common in Rift Valley (22%) and Western provinces (19%) than in the other provinces. Coast (6%), Eastern (5%) and Nyanza (5%) provinces had the highest proportions of FSW operating from sex-dens. Road (truck-stop) sex work was more common in Western province (4%) (Figure 3).

### Per capita female sex worker estimates within the towns mapped

Using gender-disaggregated population data for each of the towns mapped, we calculated the proportion of women of reproductive age who could be sex workers. We used the Kenya population census (2009) figures for the women of reproductive age (15–49 years) by town and province [17], [18]. We estimated that 5 per cent of women of reproductive age and 4 per cent of all adult women (15+ years old) in the towns mapped were sex workers. Provincial-level estimates of the proportion of women of reproductive age (15–49 years) who could be sex workers, based on data from the towns mapped, however, varied markedly, from 11 per cent in Western province to 4 per cent in Nairobi. Per capita FSW estimates also varied substantially across the individual towns mapped, with some towns having more than 20 per cent of women of reproductive age who could be sex workers: Kisii (21%), Bondo (20%), both in Nyanza province; Voi, in Coast province (22%); Webuye (20%), in Western province; and Maua (36%), Makindu (36%) and Emali (70%), all in Eastern province (Table 4 and Table 5).

**Table 4:** Per capita FSW in the towns mapped in Nairobi, Central, Eastern, Coast and Nyanza provinces, Kenya.

Province	Town	Total urban and peri-urban population	Total urban female population (15–49 years)	Total urban female population (15+ years)	Point estimate for FSW population (mapping)	% of adult women 15+ years sex workers	% of women 15–49 years sex workers
Nairobi	Westlands	247,102	58,730	85,281	2,929	3%	5%
	Kasarani	525,624	124,291	180,481	3,791	2%	3%
	Embakasi	925,775	219,685	319,002	3,878	1%	2%
	Makadara	218,641	50,008	72,616	2,482	3%	5%
	Kamukunji	261,855	59,969	87,080	2,491	3%	4%
	Starehe	274,607	63,605	92,359	6,763	7%	11%
	Dagoretti	329,577	78,329	113,741	2,433	2%	3%
	Langata	355,188	81,289	118,038	2,855	2%	4%
*Sub-total*		*3,138,369*	*735,907*	*1,068,598*	*27,620*	*3%*	*4%*
Central	Kiambu	84,155	21,076	28,101	862	3%	4%
	Nyeri	119,353	28,700	38,266	988	3%	3%
	Thika	136,917	32,884	43,846	1,933	4%	6%
	Ruiru	238,858	57,461	76,615	1,808	2%	3%
	Kerugoya	19,422	4,916	6,554	739	11%	15%
	Nyahururu	36,450	9,043	12,057	801	7%	9%
	Murang'a	28,775	7,059	9,412	442	5%	6%
*Sub-total*		*663,930*	*161,138*	*214,851*	*7,572*	*4%*	*5%*
Eastern	Embu	60,673	14,834	17,987	1,032	6%	7%
	Chuka	43,420	10,658	12,923	560	4%	5%
	Meru	53,627	13,060	15,836	1,276	8%	10%
	Maua	17,226	4,284	5,194	1,555	30%	36%
	Machakos	150,041	36,359	44,085	872	2%	2%
	Mavoko	137,211	29,930	36,291	1,973	5%	7%
	Makindu	8,621	2,189	2,654	786	30%	36%
	Emali	7,024	1,694	2,054	1,180	57%	70%
	Matuu	50,750	12,368	14,996	540	4%	4%
	Kitui	109,568	26,836	32,539	794	2%	3%
*Sub-total*		*638,161*	*152,213*	*184,558*	*10,567*	*6%*	*7%*
Coast	Mombasa	938,131	216,923	260,760	9,288	4%	4%
	Kilifi	48,826	11,994	14,417	624	4%	5%
	Mtwapa	48,625	11,756	14,131	1,118	8%	10%
	Mariakani	24,055	5,723	6,879	624	9%	11%
	Voi	17,152	4,117	4,949	900	18%	22%
	Taveta	19,865	4,712	5,664	489	9%	10%
	Malindi	118,265	28,355	34,085	2,310	7%	8%
	Ukunda	65,529	14,649	17,609	1,112	6%	8%
*Sub-total*		*1,280,448*	*298,228*	*358,495*	*16,465*	*5%*	*6%*
Nyanza	Kisumu	388,311	93,328	105,188	4,041	4%	*4%*
	Bondo	33,468	8,390	9,456	1,676	18%	*20%*
	Siaya	22,568	5,648	6,366	473	7%	*8%*
	Kisii	81,801	19,511	21,990	4,063	18%	*21%*
	Migori	53,100	13,184	14,859	2,272	15%	*17%*
	Nyamira	41,668	10,370	11,688	856	7%	*8%*
	Homa Bay	58,936	14,813	16,695	995	6%	*7%*
*Sub-total*		*679,852*	*165,243*	*186,243*	*14,375*	*8%*	*9%*

**Table 5:** Per capita FSW in the towns mapped in Western and Rift Valley provinces, Kenya.

Province	Town	Total urban and peri-urban population	Total urban female population (15–49 years)	Total urban female population (15+ years)	Point estimate for FSW population (mapping)	% of adult women 15+ years sex workers	% of women 15–49 years sex workers
Western	Kakamega	91,768	21,936	24,175	1,238	5%	*6%*
	Webuye	41,344	10,071	11,099	1,990	18%	*20%*
	Bungoma	55,867	13,535	14,917	1,994	13%	*15%*
	Busia	51,981	12,859	14,171	2,474	17%	*19%*
	Mumias	99,987	24,522	27,025	2,167	8%	*9%*
	Vihiga	118,696	29,707	32,739	2,749	8%	*9%*
	Malaba	21,477	5,261	5,798	708	12%	*13%*
*Sub-total*		*481,120*	*117,890*	*129,925*	*13,320*	*10%*	*11%*
Rift Valley	Nakuru	307,990	73,012	83,204	4,384	5%	*6%*
	Naivasha	169,142	40,457	46,104	925	2%	*2%*
	Narok	38,653	9,021	10,280	576	6%	*6%*
	Kajiado	14,860	3,502	3,990	282	7%	*8%*
	Namanga	9,066	2,103	2,397	345	14%	*16%*
	Kapenguria	34,046	8,242	9,392	1,004	11%	*12%*
	Kitale	106,187	25,019	28,511	815	3%	*3%*
	Eldoret	289,380	68,536	78,103	2,442	3%	*4%*
	Nanyuki	38,198	8,975	10,227	554	5%	*6%*
	Kericho	101,808	23,772	27,090	1,116	4%	*5%*
	Ongata Rongai	40,178	10,035	11,436	937	8%	*9%*
*Sub-total*		*1,149,508*	*272,673*	*310,734*	*13,380*	*4%*	*5%*
**Grand Total**		**8,031,388**	**1,903,293**	**2,453,404**	**103,299**	**4%**	***5%***

### Extrapolated national female sex worker population estimates

The data collected from the mapping study were then extended to the national level. As noted, Nairobi city was fully mapped, and the population of the urban centres with over 5,000 population that were mapped in the other provinces represented 72 per cent of the towns with 5,000 or more population. As indicated above, FSW size estimates from comparably-sized towns of over 5,000 population that were mapped in the other provinces were extended to North Eastern Province, which was not mapped. The extrapolated national estimate for the urban (town population over 5,000) FSW population was 138,420 (range 107,552 to 169,288). Nairobi had the largest share (20%) of the FSW population in the country, followed by Rift Valley (17%), Coast (14%), Nyanza (14%), Eastern (12%), Western (12%), Central (10%) and North Eastern (2%) provinces (Table 6).

**Table 6:** National FSW population estimates.

Province	Total pop.	Urban female pop. (15+ years)	Urban female pop. (15–49 years)	Estimated FSW pop. (mapping)	Estimated FSW population range (minimum to maximum)		% urban women (15+ years) FSW	% urban women (15–49 years) FSW
Nairobi	3,138,369	889,221	735,907	27,620	21,081	34,160	3%	4%
Central	4,383,743	491,124	368,343	13,584	10,959	16,210	3%	4%
Coast	3,325,307	415,929	346,007	19,778	15,204	24,353	5%	6%
Eastern	5,668,123	354,256	292,170	16,149	12,368	19,930	5%	6%
North Eastern	2,310,757	90,454	89,892	2,189	1,890	2,488	2%	2%
Nyanza	5,442,711	375,965	333,573	19,406	15,243	23,569	5%	6%
Rift Valley	10,006,805	645,647	566,564	23,708	18,524	28,892	4%	4%
Western	4,334,282	192,213	174,408	15,985	12,285	19,686	8%	9%
**Total**	**38,610,097**	**3,454,808**	**2,906,864**	**138,420**	**107,552**	**169,288**	**4%**	**5%**

Pop. – Population.

### Extrapolated national per capita female sex worker estimates

We also used data from the last national census to calculate the per capita number of FSW for urban areas within each province. We estimated that 4 per cent of the urban female population aged 15+ years in Kenya could be sex workers and that 5 per cent of the urban female population of reproductive age (15–49 years) in Kenya could be sex workers. There again were wide variations across provinces, ranging from 9 per cent in Western Province to 2 per cent in North Eastern Province (Table 6).

## Discussion

This study estimating the size of the population of female sex workers in Kenya was conducted in all except one of Kenya's eight former administrative provinces, and represents the single largest size estimation study to have ever been undertaken in the country. Covering a total of 51 urban centres, the study estimated the FSW population in towns with over 5,000 population at 138,420 (range 107,552–169, 288). Venue-based (mainly bar-based) sex work was the most common typology, followed by street-based sex work. We estimated that 4 per cent of the urban (towns over 5,000 population) female population aged 15+ years in Kenya and 5 per cent of the urban female population of reproductive age could be sex workers.

There have been a few other FSW population size estimation studies conducted in Kenya, covering some of the towns that were included in our geographical mapping study. In 2009, the Kenya AIDS Control Project conducted a mapping of hot spots frequented by FSW in Nairobi's Central Business District, which is a part of Starehe district, using the capture-recapture method. That study estimated the FSW population in Nairobi's Central Business District at 6,904 (95% CI 6,690–7,118). They operated mostly from fixed venues or bars (59.0%) and street-based spots (23.6%) [19]. The FSW population size estimate from that study is very close to the estimate in our study for Starehe district. In addition, the typology of sex work spots identified in this study, showing a predominance of bar-based spots in Nairobi, is consistent with the findings of our study.

In another study using a combination of multiplier and “wisdom of the crowds” methods, integrated into a behavioural surveillance survey, Okal et al. [9] estimated the population of FSW in Nairobi at 29,494 (range 10,000 to 54,467) in 2010/2011. This estimate is also close to the one in our study, with the exception of the upper bound estimate, which was based on the service multiplier technique. However, the accuracy of the service multiplier technique for population size estimation can be affected by low programme coverage and the quality of programme data.

In 2008, Vuylsteke et al. [8] estimated the population of FSW in Kisumu city, Nyanza province, using the capture-recapture method. This study was implemented with a 6-day interval between the two captures. The majority (87%) of the sex workers were identified from bars, clubs and dance venues. This study estimated the FSW population in Kisumu city at 1,350 (95% CI 1,261–1,443). However, Vuylsteke et al. did not specify the geographic coverage of their study. Our mapping study, which captured multiple sex work spots and included Kisumu city and its suburbs, estimated the total FSW population in the city at 4,041 (range 3,228–4,854).

Another large study that estimated FSW population sizes in Kenya focused on the busy transport corridor linking Mombasa, the main port city in Kenya and East Africa, with neighbouring East and Central African countries [20], [21]. This study focused on spots frequented by long-distance truck drivers for overnight stay, and as such, excluded major towns, which are generally not preferred by truckers for stop-over due to lack of parking space and concerns about costs and security. The methods applied to estimate FSW population sizes consisted of focus group discussions at some of the hot spots, including questioning on the number of sex workers at the sites, and a survey of bars and lodgings at the sites, including questioning on the presence and proportion of FSW among the clientele. From this work, the study team generated FSW population size estimates for some of the towns that were also included in our geographical mapping study. This study, for example, estimated 250 FSW in Mariakani town in Coast province, as compared to our estimate of 624 (range 478 to 769). They also estimated 100 FSW in Naivasha town, Rift Valley province and 300 in Malaba town, Western province, which compares to 925 (range 568 to 1,282) and 708 (range 554 to 862), respectively, in our study. The town with the largest number of FSW in the truck driver study was Busia (1,500), which in our study had an estimated 2,474 (range 1,854 to 3,094) FSW. Our geographical mapping study captured multiple typologies of sex work spots, many of which may have been omitted in the truck driver study. Indeed, our study showed that home-based sex workers comprised a relatively high percentage of all sex workers in Rift Valley (22%) and Western (19%) provinces.

A comparison of per capita estimates that relate the estimated FSW population size to the total adult population or the population of women of reproductive age (15–49 years) is also useful for determining the validity of population size estimates. The estimate from our study of 4 per cent of the Nairobi female population of reproductive age (15–49 years) potentially being sex workers is consistent with findings from previous studies in other major cities in sub-Saharan Africa. A study in Ouagadougou in Burkina Faso, West Africa, in 2000/2003 estimated 4.3 per cent of the adult female population in that city to be sex workers, while a study in 2002 in Addis Ababa, the capital of Ethiopia, and in Niamey, the capital of Niger in 2004, estimated 2.9 per cent and 2.1 per cent, respectively [22], [23]. A study in a provincial town in Madagascar in 2001 [24] estimated the percentage of all women in that town who could be sex workers to be 12 per cent.

The per capita FSW estimates for some of the towns mapped in our study were higher than those reported in the literature for other towns and cities in sub-Saharan Africa [22]. The high per capita FSW estimates for these towns in our study could be linked to their location and economic profile. For example, Emali and Makindu are small towns located along the northern transport corridor linking the port city of Mombasa in Kenya with neighbouring countries in East and Central Africa. These towns are primarily truck-stop centres, and it is conceivable that a large proportion of women in those towns could be involved in sex work. These results underscore the heterogeneity of the HIV epidemic at both national and sub-national levels in Kenya, and the importance of understanding local epidemic drivers in the design of effective HIV responses.

### Limitations

There are several limitations of the geographical mapping approach that we used. First, because the methodology began by identifying spots frequented by FSW through secondary key informants, there is the possibility of missing some spots and either over- or under-estimating some groups of FSW, depending on the extent of their visibility [12]. For example, sex workers who primarily contact clients through cell phones or network operators, or who are based at home, are likely to be under-represented in geographically based mapping. Second, the geographical mapping methodology relies on numeric estimates rather than a count of FSW at the spots identified, which may lead to variability in the estimates derived. Even though the methodology addresses this limitation through averaging estimates for spots identified by a large number of secondary key informants, and validating estimates for spots identified by the least number of secondary key informants through interviews with the FSW themselves, it is possible that some secondary and primary key informants may still over- or under-estimate FSW numbers. Third, since the methodology is not individually based, it could overestimate the size of FSW if FSW frequent multiple locations. For example, if a female sex worker works in multiple bars, it is possible that the same FSW could contribute to estimated numbers at multiple spots, thereby inflating the estimates. However, since the methodology is rapid and focuses on the minimum and maximum number of FSW at a spot on a given day, the range of estimates (minimum to maximum) is unlikely to be skewed substantially. Moreover, the final population size estimates derived were adjusted for mobility of FSW, based on information provided by the FSW themselves.

Another limitation of our approach is that the tools used for geographical mapping and size estimation are kept short to enhance response rates among both secondary and primary key informants. Consequently, detailed data on HIV-risk behaviour, and access to and utilisation of HIV preventive services among FSW, were not collected as part of the mapping process. The mapping data, however, provide a valid sampling frame of spots frequented by FSW, which can be used to design and undertake behavioural surveys. Building upon this geographical mapping study, we are currently undertaking a follow-up ethnographic study in Nairobi to better understand the different sex work spot typologies, how sexual networks form at these spots, and the overlap between sexual networks that are mediated by financial and other material resources. We are also undertaking a behavioural survey to assess differences in socio-demographic and economic profiles and sexual behaviours of FSW according to the type of spots from which they operate (both fixed venues and other types of spots), in order to identify unmet needs for HIV preventive services by sex work spot typology.

### Conclusions

This enumeration of FSW in Kenya provides an important starting point for both macro- and micro-level planning of HIV prevention programmes, including the prioritization of cities/towns and locations for establishing such programmes, determining the initial volume of services required, and coordinating the provision of HIV prevention programmes for FSW throughout the country. Our study represents the first national-level mapping of FSW to be conducted in Kenya. A key advantage of this approach is that it is transparent, making it possible for stakeholders implementing programmes with FSW to continuously update the estimates provided. The mapping data can be used in FSW programmes for a variety of purposes, including: registration of FSW for programme planning purposes; identification and allocation of peer educators in programme sites; and project evaluation. Programme coordination is also a challenge in Kenya, as different organisations funded by different donor agencies implement FSW programmes, sometimes overlapping within the same locality. These new data will allow the newly decentralised county governments in Kenya to play a greater role in FSW programme planning and implementation. Indeed, these data are already being used by the Kenya Ministry of Health's National AIDS/STI Control Programme to monitor the coverage and quality of existing HIV prevention programmes for female sex workers throughout the country.
